# Validation of the Croatian Version of the Eight-Item Chronic Pain Acceptance Questionnaire (CPAQ-8)

**DOI:** 10.3390/ijerph23020145

**Published:** 2026-01-23

**Authors:** Iva Dimitrijević, Ivan Radoš, Dijana Hnatešen, Vanja Matković, Dino Budrovac

**Affiliations:** 1 Department of Pain Management, University Hospital Osijek, 31000 Osijek, Croatia; iva.dimitrijevic@kbco.hr (I.D.); dijana.hnatesen@kbco.hr (D.H.); vanja.matkovic@kbco.hr (V.M.); dino.budrovac@mefos.hr (D.B.); 2 Faculty of Medicine, Josip Juraj Strossmayer University of Osijek, 31000 Osijek, Croatia; 3 Nursing Institute “Professor Radivoje Radić”, Faculty of Dental Medicine and Health Osijek, Josip Juraj Strossmayer University of Osijek, 31000 Osijek, Croatia; 4 Department of Physiotherapy, College of Applied Sciences “Lavoslav Ružička”, 32000 Vukovar, Croatia

**Keywords:** chronic pain, acceptance, chronic pain acceptance questionnaire, psychometric properties

## Abstract

Background: Chronic pain is a serious health issue associated with significant functional and emotional impairment. Chronic pain acceptance, which can be described as engaging in valued activities despite ongoing pain, is associated with better psychological adjustment and quality of life. This study aimed to translate the Eight-Item Chronic Pain Acceptance Questionnaire (CPAQ-8) into Croatian and investigate its reliability and validity. Methods: A total of 229 outpatients with chronic musculoskeletal pain completed the Croatian version of the CPAQ-8, as well as the SF-36 Health Status Questionnaire (SF-36), the Hospital Anxiety and Depression Scale (HADS), the Pain Catastrophizing Scale (PSC), and the Numeric Pain Rating Scale (NRS). Results: The Croatian version of the CPAQ-8 demonstrated good internal consistency for the total score and its subscales, and the exploratory factor analysis revealed the original two-factor structure. Concurrent validity was supported through theoretically consistent correlations with psychological distress, quality of life, and pain-related constructs. Conclusion: The Croatian version of the CPAQ-8 is a reliable and valid instrument for assessing pain acceptance in patients with chronic musculoskeletal pain and can be confidently used in both clinical practice and research settings.

## 1. Introduction

Chronic pain, which can be defined as pain that lasts or recurs for more than three months [1], is a complex and ongoing health condition with a worldwide prevalence of 20%. It is a serious health issue that is associated with significant functional impairment, including high disability days [2], poorer quality of life [3,4], higher psychological distress [3], anxiety, and depression [5,6]. Given the numerous recognized difficulties that occur as part of chronic pain, the importance of a biopsychosocial approach in the research and treatment of chronic pain is recognized. According to the biopsychosocial approach, chronic and complicated pain syndromes are the result of a complex and dynamic interplay between physiological, psychological, and social elements that sustain and even exacerbate one another [7]. For a long time, psychological factors such as anxiety [8,9], depression [9,10], pain catastrophizing [11,12], and kinesiophobia [13] have attracted the attention of researchers, and a connection between these factors and chronic pain has been found [14]. However, recently, newer constructs such as chronic pain acceptance have proven to be significant in the treatment and study of chronic pain [15]. The concept of chronic pain acceptance can be described as the willingness to experience ongoing pain without attempts to avoid, reduce, or control it and engage in the activities of daily life despite the experience of chronic pain [16]. Recent studies support that higher pain acceptance is associated with lower pain intensity, better quality of life, and reduced psychological distress [17]. Pain acceptance is most often assessed with the Chronic Pain Acceptance Questionnaire (CPAQ) [18].

Geisser first created the Chronic Pain Acceptance Questionnaire (CPAQ) in an unpublished doctoral dissertation, originally developed in English. It was later revised by McCracken, Vowles, and Eccleston, who recommended a 20-item version with two subscales: activity engagement, which refers to the degree to which one engages in life activities regardless of pain, and pain willingness, which represents willingness to experience pain, which is the opposite of engaging in behaviors to limit contact with pain [19]. It is a commonly used tool in research and treatment because numerous studies have demonstrated its strong psychometric qualities [20], and it is a good predictor of disability, quality of life, and psychological distress [19]. Later, based on a statistical analysis of items from an internet sample, an eight-item version of the CPAQ, called the CPAQ-8, was developed [21]. It showed a high correlation with the 20-question version, with an advantage in brevity, resulting in a briefer administration [21,22]. Based on its benefits, the scale has been translated into numerous languages [22,23,24] and is often used in research and clinical practice. The CPAQ-8, like its original version, also showed good psychometric properties and the same two-factor structure [18,21,22,23].

Despite all the aforementioned advantages and benefits of using the CPAQ-8 in research and clinical practice, unfortunately, a Croatian version of the questionnaire has not yet been developed. Developing culturally adapted instruments is essential to ensure that assessments accurately capture patients’ experiences and are sensitive to linguistic and cultural nuances. Therefore, this study aims to translate the CPAQ-8 into Croatian and investigate its reliability and validity in a Croatian sample of patients with chronic pain. The goal of translating and validating the aforementioned scale is to increase the frequency of research on chronic pain as a significant public health issue. Furthermore, by expanding the database of Croatian validated questionnaires related to chronic pain, we want to encourage their more frequent use in the treatment process and the evaluation of various therapeutic procedures. A validated Croatian CPAQ-8 can be applied widely in clinical practice, supporting physicians, clinical psychologists, physiotherapists, occupational therapists, and other healthcare professionals in monitoring treatment outcomes and tailoring interventions to individual patient needs.

## 2. Materials and Methods

### 2.1. Participants

The participants included 229 outpatients with chronic musculoskeletal pain from the Clinical Department of Pain Management at the University Hospital Osijek, Croatia. Participants were recruited using convenience sampling with consecutive inclusion of all patients referred for a specialist pain management examination who met the inclusion criteria and agreed to participate. The sample size was determined using the COSMIN risk of bias checklist, which specifies a required sample size of 7 times the number of questionnaire items and greater than 100 participants [25]. Inclusion criteria required participants to be 18 years or older, to have experienced musculoskeletal pain for at least three months, and to report pain intensity of 3 or higher on the Numeric Rating Scale (NRS). Exclusion criteria included acute pain, cancer-related pain, psychotic disorders, moderate to severe cognitive impairment, and post-traumatic conditions. Physicians assessed the absence of psychosis and cognitive deficits during clinical evaluation and review of medical records. All participants provided written informed consent. The study was approved by the institutional Ethics Committee (R1-11662-2/2022) and conducted in accordance with the principles outlined in the Declaration of Helsinki.

### 2.2. Measures

A sociodemographic data form collected information on participants’ age, sex, location of pain, residence, education level, working status, and marital status.

The SF-36 Health Status Questionnaire (SF-36) was used to evaluate health-related quality of life. The SF-36 questionnaire represents two general concepts of health: physical health (PhyH) and psychological health (PsyH) dimensions of Health-Related Quality of Life (HRQoL). The Physical Health (PhyH) dimension includes the following subscales: physical functioning, bodily pain, role limitations due to physical health, and general health. The psychological health (PsyH) dimension includes the following subscales: vitality, social functioning, role limitations due to emotional problems, and mental health. On a representative sample of Croatian adults, the SF-36 questionnaire showed strong psychometric qualities [26]. For every subscale in the current study, Cronbach’s alpha was acceptable. The general health subscale had the lowest Cronbach’s alpha of 0.72, while the role limitations due to emotional difficulties subscale had the highest Cronbach’s alpha of 0.91.

The Hospital Anxiety and Depression Scale (HADS) was used to measure anxiety and depression. There are 14 items on the scale, 7 of which are associated with anxiety and 7 with depression. With Cronbach’s alpha values for the subscales ranging from 0.68 to 0.93 for anxiety and from 0.67 to 0.90 for depression, the HADS showed strong psychometric qualities [27]. A two-factor structure was also found in the Croatian version of HADS, and the scales demonstrated an excellent internal consistency and good convergent validity [28]. Cronbach’s alpha for anxiety and depression in the current study was 0.88 and 0.83, respectively.

The Pain Catastrophizing Scale (PCS) was used to measure catastrophizing associated with the pain experience. Three subscales—rumination, exaggeration, and helplessness—are included in its thirteen items, with a total score used due to strong intercorrelations among subscales [29]. The Croatian version has demonstrated a comparable factor structure and good internal consistency [30]. In the present study, internal consistency was excellent (Cronbach’s alpha = 0.95).

The Numeric Pain Rating Scale (NRS) was used to assess pain intensity. The scale consists of a solid line with numbers ranging from 0 to 10 at either end. The absence of pain is represented by the number 0 on the far left, and excruciating pain is represented by the number 10 on the far right [31].

The Chronic Pain Acceptance Questionnaire-8 (CPAQ-8) was used to evaluate the acceptance of chronic pain. Specific features of pain acceptance are measured by the two subscales of the CPAQ-8 questionnaire. The Activity Engagement (AE) measure assesses respondents’ perceived level of activity despite their ongoing pain. The measure of Pain Willingness (PW) assesses respondents’ willingness to tolerate pain without resorting to inefficient pain treatment strategies. Lower scores on a Likert scale (0–6) indicate lower levels of AE and PW [32]. All of the CPAQ-8 scales demonstrated good internal consistency, with Cronbach’s alpha greater than 0.80 [18]. The study followed the widely cited guidelines for cross-cultural adaptation of self-report measures [33] to ensure equivalence between the original English version and the Croatian version. Permission was obtained from the original authors to translate and validate the CPAQ-8. The scale was translated into Croatian by two bilingual translators and reviewed by the research team to select the most appropriate and culturally relevant expressions. A separate two translators who were not involved in the initial translation process performed a back-translation, which was compared to the original version to identify discrepancies. The finalized version was piloted with 10 patients with chronic pain to assess clarity, comprehensibility, and cultural relevance. Based on participant feedback, minor wording adjustments were made to improve clarity, resulting in the final version used in the present study. The current study’s PW and AE have respective Cronbach’s alphas of 0.74 and 0.83.

### 2.3. Data Analysis

IBM SPSS Statistics (version 24.0.0.0; IBM Corp., 2016; IBM SPSS Statistics for Windows, IBM Corp., Armonk, NY, USA) software tools were used for statistical analysis, and *p* < 0.05 was considered statistically significant. Data normality was evaluated using skewness and kurtosis values, which indicated no substantial deviations from normality. Consequently, parametric analyses were conducted, and Pearson’s correlation coefficients were used.

The Cronbach’s alpha method was used to evaluate the internal consistency. The homogeneity of the items is shown in Cronbach’s alpha, which was computed for both the overall score and each subscale independently. A Cronbach’s alpha value of 0.7 is considered the minimum requirement for internal consistency. Although the original CPAQ-8 has a well-established two-factor structure, exploratory factor analysis was conducted to examine whether the same factor structure would emerge in the Croatian cultural and linguistic context. Construct validity was assessed by comparing the CPAQ-8 total and subscale scores with those of other specified scales.

## 3. Results

The demographic parameters of the participants are presented in Table 1. A total of 229 patients who met the inclusion criteria, consented to participate, signed the informed consent form, and completed the questionnaires were included in this study. The age range was 27 to 82 years, with a mean of 54.21 +/− 12.088 years. 76.9% of the sample were female, 65.7% lived in cities, 62.1% were employed, 63.8% were married, 69.0% had completed secondary school, and 50.7% had pain for longer than seven years.

The internal consistency of the CPAQ-8 was measured with Cronbach’s alpha. Table 2 shows the Cronbach’s alpha values for the total score of the questionnaire and for the two subscales of the questionnaire. Cronbach’s alpha values for AE and PW subsales were found to be 0.86 and 0.73, respectively.

The results of the item statistics are summarized in Table 3. The arithmetic mean of individual items ranges from 3.93 to 4.64. The corrected item total correlation value, accounting for item overlap, is also shown, ranging from 0.385 to 0.696.

The exploratory factor analysis revealed a clear two-factor solution consistent with the original CPAQ-8 structure. Before conducting the factor analysis, the suitability of the data was assessed. The results showed that the Kaiser–Meyer–Olkin measure of sampling adequacy was 0.811, indicating good adequacy for factor analysis. Bartlett’s test of sphericity was statistically significant (χ^2^ = 768.93, df = 28, *p* < 0.001), confirming that the correlation matrix is not an identity matrix and that the data are appropriate for factor extraction. The results of the exploratory factor analysis are presented in Table 4 and Table 5. Table 4 includes eigenvalues, the percentage of variance explained by each factor, and the cumulative variance explained, and Table 5 includes the rotated factor loadings, obtained using the Varimax rotation method with Kaiser normalization.

Concurrent criterion validity was analyzed using Pearson’s correlation coefficient. Table 6 shows the correlations between the CPAQ-8 total and the CPAQ-8 subscales with the criterion variables. CPAQ-8 total had a weak but statistically significant correlation with PSC, a moderate statistically significant correlation with HADS-A, HADS-D, SF-Phy, and SF-Psy. No significant correlation was found between CPAQ-8 total and NRS, and CPAQ-8 PW had a weak, statistically significant correlation with PSC. However, no significant correlations were found between CPAQ-8PW and HADS-A, HADS-D, SF-Phy, SF-Psy, and NRS. Finally, the CPAQ-8AE showed a weak statistically significant correlation with NRS and moderate statistically significant correlations with HADS-A, HADS-D, SF-Phy, and SF-Psy.

## 4. Discussion

An increasing amount of empirical research indicates that coping with chronic pain is significantly influenced by pain acceptance [24]. The present study aimed to translate and validate the Croatian version of the Chronic Pain Acceptance Questionnaire-8 (CPAQ-8) in a sample of patients with chronic musculoskeletal pain. Overall, the findings support the reliability and validity of the Croatian CPAQ-8 and are in line with previous validation studies conducted in other languages and cultural settings.

The Croatian CPAQ-8 demonstrated good internal consistency, with a Cronbach’s alpha of 0.83 for the total scale, 0.86 for the AE subscale, and 0.73 for the PW subscale. These values fall within the acceptable to excellent range and are comparable with previous CPAQ-8 validations, including the original English version [21], as well as Thai [34], Japanese [23], and Turkish [35] versions. In line with earlier studies, the AE subscale showed stronger internal consistency than the PW subscale. Across validations, the PW subscale consistently demonstrates slightly lower reliability [21,23,24,34,35], likely reflecting the more heterogeneous content and reverse-scored nature of PW items noted in prior research by Fish et al. [21].

A corrected item–total correlations ranged from 0.385 to 0.704, all above the recommended minimum of 0.30. These results confirm good internal homogeneity of the Croatian items and support the conceptual coherence of the two CPAQ-8 subdomains. Notably, items within the AE subscale exhibited particularly strong correlations (0.581–0.704), whereas PW items showed comparatively lower values, a pattern consistent with previous CPAQ-8 validation studies [34].

The exploratory factor analysis revealed a two-factor structure that reflected AE and PW, which is consistent with the original instrument [21] and other validation studies [23,24,33,34]. The Kaiser–Meyer–Olkin value of 0.811 and a significant Bartlett’s test confirmed the suitability of the data for factor analysis. The two extracted components accounted for 65.5% of the total variance, which is comparable to reports from Japanese (68%) [23], Turkish (63%) [35], and Thai (66%) [34] samples. The theoretical model underlying pain acceptance, which involves both participation in meaningful activities and readiness to feel pain without excessive attempts at control, was further supported by item loadings that were in good agreement with the expected factor configuration.

Construct validity of the Croatian CPAQ-8 was supported through correlations with measures of pain intensity, emotional distress, pain catastrophizing, and quality of life. As expected, higher acceptance was associated with lower levels of anxiety, depression, and catastrophizing, as well as better physical and psychological functioning. However, the strength of these associations ranged from weak to moderate. These results are consistent with those of other validation studies [18,23,24,34,35], which also demonstrated modest but significant relationships between pain acceptance and indicators of emotional well-being and functional health. The AE subscale showed relatively stronger associations with clinical variables, particularly emotional distress and quality of life suggesting that this component may be especially relevant for understanding behavioral adaptation to chronic pain. This result is consistent with the validation results of the Japanese [23] and Swedish [18] versions of the CPAQ-8 and may indicate that this subscale is especially relevant in capturing behavioral adaptation to chronic pain. Conversely, the PW subscale showed weaker and more inconsistent correlations, warranting deeper exploration. Cultural and linguistic factors may contribute to these results, as items assessing willingness to experience pain without attempts to control or avoid it can be interpreted differently across cultural contexts. In Croatian cultural contexts, where stoicism and pain endurance are culturally valorized through historical resilience narratives, respondents might endorse PW items uniformly high. This would attenuate variability and reliability. Due to linguistic factors, expressions related to “enduring” or “accepting” pain may carry negative connotations or be cognitively demanding, potentially leading to increased response variability. Taken together, these findings highlight the importance of continued cross-cultural research aimed at improving the conceptual clarity and cultural sensitivity of pain willingness items. It is interesting to note that there was no significant correlation between the overall CPAQ-8 score, PW subscale, and pain intensity, and a low correlation was found between AE subscale and pain intensity. A similar result was obtained in the validation of the Thai version of the CPAQ-8 [34], while some other validations obtained a significant correlation between the CPAQ-8 and pain intensity [18,22,23]. These results should be interpreted cautiously, as they indicate small effect sizes. Nonetheless, they are consistent with the theoretical framework of pain acceptance, which emphasizes changes in coping and psychological flexibility rather than reductions in perceived pain intensity, underscoring the conceptual distinction between pain severity and adaptive functioning in chronic pain. Although divergent validity was not formally assessed using theoretically unrelated constructs, the weak or non-significant correlations with pain intensity are consistent with previous findings [22,24,34] and support the conceptual distinction between pain acceptance and pain severity.

There are a number of significant benefits to having a validated Croatian version of the CPAQ-8. In clinical settings, it provides practitioners such as clinical psychologists, pain specialists, and multidisciplinary team members with a brief and reliable tool for assessing patients’ levels of pain acceptance. This facilitates individualized treatment planning by identifying patients with low acceptance who may benefit from targeted acceptance and commitment therapy (ACT) or cognitive-behavioral therapy (CBT) interventions that emphasize values-based action despite pain. For outcome evaluation, the CPAQ-8 supports repeated, low-burden assessments to track changes in pain acceptance over time, allowing clinicians to quantify intervention efficacy. For instance, pre- and post-treatment scores can gauge responsiveness to ACT or CBT interventions. In research, it contributes to the cross-cultural comparability of studies examining acceptance-based constructs and supports further investigation into the role of psychological flexibility in chronic pain within Croatian populations, potentially informing public health policies.

Despite its strengths, the study has certain limitations. All participants were recruited from a single tertiary pain clinic in Osijek, which may limit the generalizability of findings to other clinical settings, regions, and populations with non-musculoskeletal chronic pain or with a different etiology or primary cause of pain. Onwards, the cross-sectional design did not allow for assessment of test–retest reliability or responsiveness to treatment over time. Future longitudinal studies should examine the stability of the Croatian version of the CPAQ-8 over time and its sensitivity to therapeutic change. Furthermore, the study relied exclusively on self-report measures, which may be subject to response biases such as social desirability, recall bias, or differences in individual interpretation of questionnaire items. Although self-report instruments are commonly used and appropriate for assessing subjective pain-related constructs, these factors should be considered when interpreting the results. Future research could benefit from incorporating complementary assessment methods, such as clinician-rated measures or behavioral indicators, to further strengthen validity.

## 5. Conclusions

The Croatian version of the CPAQ-8 demonstrated good reliability, a stable two-factor structure, and satisfactory construct validity, consistent with findings from other international validations. These results support the use of the Croatian version of the CPAQ-8 as a valid tool for assessing pain acceptance in individuals with chronic musculoskeletal pain. Its inclusion in clinical and research contexts may enhance understanding of psychological processes in chronic pain and promote the integration of acceptance-based approaches within Croatian pain management practice.

## Figures and Tables

**Table 1:** Patients’ sociodemographic characteristics.

Characteristics	Value
Gender, n (%)	
Male	53 (23.1)
Female	176 (76.9)
Age (years), mean +/− SD	54.21 +/− 12.088
Residence, n (%)	
Urban	151 (65.9)
Rural	78 (34.1)
Working status, n (%)	
Employed	142 (62.1)
Unemployed	29 (12.7)
Retired	58 (25.3)
Marital status, n (%)	
Married	146 (63.8)
Divorced	69 (30.1)
Single	9 (3.9)
Widowed	5 (2.2)
Education level, n (%)	
Primary education	17 (7.4)
Secondary education	158 (69.0)
Tertiary education	54 (23.6)
Pain duration	
<1 year	44 (19.2)
1–3 years	37 (16.2)
4–6 years	32 (14.0)
>7 years	116 (50.7)

**Table 2:** Cronbach’s alpha values of the Eight-Item Chronic Pain Acceptance Questionnaire (CPAQ-8) and CPAQ-8 subscales.

Variables	Cronbach’s Alpha	No. of Items
CPAQ-8Total	0.83	8
CPAQ-8PW	0.73	4
CPAQ-8AE	0.86	4

CPAQ-8Total—total score of the Croatian version of the Chronic Pain Acceptance Questionnaire; CPAQ-8PW—Pain Willingness subscale; CPAQ-8AE—Activity Engagement subscale.

**Table 3:** Item statistics for the CPAQ-8.

Item	Mean	Standard Deviation	Minimum	Maximum	Corrected ITC
CPAQ-1	4.64	1.131	1	6	0.645
CPAQ-2	4.52	1.009	3	6	0.611
CPAQ-3	4.26	1.338	0	6	0.696
CPAQ-4	4.51	0.927	3	6	0.504
CPAQ-5	3.95	1.563	0	6	0.704
CPAQ-6	3.93	1.528	3	6	0.581
CPAQ-7	4.62	0.970	3	6	0.456
CPAQ-8	4.38	1.090	3	6	0.385

Corrected ITC—corrected item-total correlations.

**Table 4:** Exploratory factor analysis eigenvalues and explained variance.

Component	Eigenvalue	% of Variance	Cumulative %
1. CPAQ-8AE	3.73	46.68	46.68
2. CPAQ-8PW	1.51	18.86	65.54

CPAQ-8PW—Pain Willingness subscale; CPAQ-8AE—Activity Engagement subscale.

**Table 5:** Factor loadings of the CPAQ-8 items.

Item	CPAQ-8AE	CPAQ-8PW
CPAQ-1	0.643	0.406
CPAQ-2	0.434	0.658
CPAQ-3	0.872	0.161
CPAQ-4	0.199	0.817
CPAQ-5	0.877	0.170
CPAQ-6	0.848	0.024
CPAQ-7	0.255	0.619
CPAQ-8	−0.121	0.763

CPAQ-8PW—Pain Willingness subscale; CPAQ-8AE—Activity Engagement subscale.

**Table 6:** Correlations between CPAQ-8 and pain intensity, anxiety, depression, physical and psychological dimensions of quality of life, and pain catastrophizing.

	CPAQ-8	CPAQ-8PW	CPAQ-8AE	NRS	HADS-A	HADS-D	SF-Phy	SF-Psy	PSC
CPAQ-8	1	0.76 **	0.91 **	−0.07	−0.39 **	−0.48 **	0.38 **	0.35 **	−0.21 **
CPAQ-8 PW		1	0.42 **	0.09	−0.06	−0.11	0.02	−0.01	0.15 *
CPAQ-8 AE			1	−0.15 *	−0.50 **	−0.60 **	0.52 **	0.49 **	−0.38 **
NRS				1	0.19 **	0.21 **	−0.49 **	−0.35 **	0.35 **
HADS-A					1	0.80 **	−0.62 **	−0.071 **	0.61 **
HADS-D						1	−0.066 **	−0.68 **	−0.53 **
SF-Phy							1	0.75 **	−0.51 **
SP-Psy								1	−0.051 **
PCS									1

CPAQ-8—Total score of the Croatian version of the Chronic Pain Acceptance Questionnaire; CPAQ-8PW—Pain Willingness subscale; CPAQ-8AE—Activity Engagement subscale; NRS—the Numeric Pain Rating Scale; HADS-A—the Hospital Anxiety and Depression Scale—Anxiety subscale; HADS-D—the Hospital Anxiety and Depression Scale—Depression subscale; SF-Phy—The SF-36 Health Status Questionnaire—the Physical Health dimension; SF-Psy—the SF-36 Health Status Questionnaire—the Psychological Health dimension; PCS—the Pain Catastrophizing Scale; *—*p* < 0.05, **—*p* < 0.01.

## Data Availability

The data presented in this study are available upon request from the corresponding author.

## Abbreviations

The following abbreviations are used in this manuscript:

CPAQ	Chronic Pain Acceptance Questionnaire
CPAQ-8	Eight-Item Chronic Pain Acceptance Questionnaire (CPAQ-8)
HRQoL	Health-related quality of life
SF-36	The SF-36 Health Status Questionnaire
SF-Phy	Physical health dimension of HRQoL
SF-Psy	Psychological health dimension of HRQoL
HADS	The Hospital Anxiety and Depression Scale
HADS-A	The Hospital Anxiety and Depression Scale—Anxiety subscale
HADS-D	The Hospital Anxiety and Depression Scale—Depression subscale
PCS	The Pain Catastrophizing Scale
